# Study on the application of prone and supine lung recruitment maneuvers in the treatment of atelectasis after minimally invasive direct coronary artery bypass surgery

**DOI:** 10.3389/fsurg.2025.1665139

**Published:** 2025-10-02

**Authors:** Yue Xin, Zelin Meng, Zhou Fu, Pixiong Su

**Affiliations:** 1 Department of Cardiovascular Surgery, Beijing Chao-Yang Hospital, Capital Medical University, Beijing, China; 2 Department of Hepatobiliary Surgery, Huairou Hospital Beijing Chao-yang Hospital, Capital Medical University, Beijing, China

**Keywords:** minimally invasive direct coronary artery bypass grafting (MIDCABG), atelectasis, lung recruitment strategy, prone position, oxygenation index

## Abstract

**Background:**

Atelectasis is a common complication after minimally invasive direct coronary artery bypass grafting (MIDCABG), which can lead to hypoxemia and even life-threatening conditions. This study aimed to compare the efficacy of prone vs. supine lung recruitment maneuvers in patients undergoing MIDCABG.

**Methods:**

This retrospective study included 170 patients who underwent MIDCABG and developed hypoxemia due to atelectasis during postoperative invasive mechanical ventilation in the cardiac surgical intensive care unit (CSICU). Patients were randomized into prone and supine groups. Clinical recovery indicators and physiological and laboratory parameters at different time points were compared between the two groups. Multiple linear regression was used to analyze the effect of different lung recruitment strategies on the improvement of the oxygenation index. Subgroup analyses were conducted to assess whether the effect of prone vs. supine lung recruitment on oxygenation improvement varied across different patient populations.

**Results:**

Prone-position lung recruitment significantly reduced hospital stay, extubation time, time to first ambulation, time to first flatus, duration of mechanical ventilation, and duration of non-invasive oxygen therapy. Lung recruitment strategies significantly improved oxygenation index, carbon dioxide elimination, heart rate control, and inflammatory markers, with the prone group showing superiority at multiple key time points. Multiple linear regression indicated that the prone lung recruitment strategy significantly enhanced the improvement of the oxygenation index, and this effect remained robust after adjusting for age, sex, BMI, and baseline comorbidities. Subgroup analyses revealed that the beneficial effect of prone lung recruitment was more pronounced in patients without diabetes and those with a history of PCI.

**Conclusion:**

Lung recruitment significantly improves oxygenation, carbon dioxide clearance, heart rate control, and inflammatory markers in MIDCABG patients with postoperative atelectasis, with the prone strategy being more effective than the supine. Multivariable and subgroup analyses confirmed the robustness of this effect, particularly in non-diabetic patients and those with a history of PCI.

## 1 Introduction

Coronary heart disease (CHD) is a type of cardiovascular disease caused by atherosclerosis, narrowing, or occlusion of the coronary arteries, resulting in myocardial ischemia, hypoxia, or even infarction (1, 2). It has become a major threat to public health in China (3), and improving the treatment of CHD is an urgent priority in cardiovascular medicine. Current clinical treatments for CHD include medical therapy, percutaneous coronary intervention (PCI), and surgical revascularization. Among them, coronary artery bypass grafting (CABG), which achieves myocardial revascularization by bypassing occluded coronary segments, remains the primary strategy for patients with multivessel coronary disease (4). Various CABG approaches are available, including conventional on-pump CABG, off-pump CABG (OPCABG) (5), minimally invasive direct CABG (MIDCABG), minimally invasive multivessel CABG (MICS-CABG) (6), and robot-assisted CABG (RACAB) (7).

Although conventional on-pump CABG offers a clear surgical field and effective revascularization, it is associated with significant surgical trauma and potential complications such as systemic inflammatory responses, microthrombi formation, and postoperative respiratory, neurologic, and transfusion-related issues. With advancements in surgical exposure and stabilization techniques, MIDCABG—offering smaller incisions and shorter hospital stays—has gained popularity in cardiac surgery (8). However, it has limitations in terms of patient selection and intraoperative single-lung ventilation, which may adversely affect pulmonary function. To address these limitations, hybrid coronary revascularization (HCR) has emerged, combining MIDCABG with PCI (9). This approach achieves LIMA-to-LAD anastomosis and PCI-based revascularization of non-LAD vessels without the use of cardiopulmonary bypass, merging the long-term benefits of surgical grafts with the minimal invasiveness of interventional therapy, and reducing trauma associated with median sternotomy and manipulation of the ascending aorta (10). Atelectasis and its resultant hypoxemia are common postoperative complications following MIDCABG (11). Postoperative atelectasis often triggers pulmonary inflammation and may be associated with perioperative ventilator-induced lung injury (12). This pathophysiological process is frequently accompanied by hypoxemia, pneumonia, ventilator-associated lung injury, and even acute respiratory distress syndrome (ARDS). Such complications may prolong invasive mechanical ventilation, increase hospital length of stay, and negatively affect prognosis.

Lung recruitment maneuvers (RMs) have been shown to increase functional residual capacity in ARDS patients, optimize ventilation/perfusion (V/Q) matching, and improve oxygenation, thereby contributing to more stable and effective patient outcomes (13). Consequently, lung recruitment techniques may offer substantial clinical benefits for patients undergoing cardiac surgery. First, they may reduce postoperative pulmonary complications and facilitate more stable recovery. Second, by reopening collapsed alveoli, RMs can alleviate intrapulmonary shunting and refractory hypoxemia, thereby improving overall ventilation. The prone position is typically considered to be more effective in promoting lung recruitment. Physiologically, the prone position improves the ventilation-perfusion matching of the lungs, increases ventilation in the lower lung regions, reduces alveolar collapse, and optimizes the oxygenation index (PaO_2_/FiO_2_) (14). Compared to the supine position, the prone position distributes pressure more evenly across the lungs, alleviates pressure accumulation in the chest cavity, particularly in the lower lung regions, and reduces the occurrence of atelectasis. While previous studies have investigated the use of RMs in cardiac surgical patients or during single-lung ventilation (15), no studies to date have compared prone vs. supine lung recruitment strategies specifically in the MIDCABG postoperative setting, but only after other cardiac surgery (16). Therefore, this study aims to compare the effects of prone vs. supine lung recruitment maneuvers on oxygenation, cardiopulmonary physiological parameters, inflammatory markers, and clinical recovery times. We hypothesised that prone recruitment vs. supine is superior in terms of oxygenation.

## 2 Materials and methods

### 2.1 Study population

Inclusion criteria were: Age over 18 years; Postoperative admission to the intensive care unit (ICU); Development of atelectasis during invasive mechanical ventilation via endotracheal intubation. Exclusion criteria were: Age <18 or >80 years; Pre-existing or newly diagnosed pulmonary diseases (defined as a history of or preoperative examination indicating airway obstruction, i.e., a forced expiratory volume in one second to forced vital capacity ratio [FEV1/FVC] <70%); Hemothorax or massive pleural effusion detected by ultrasound or radiographic imaging; History of cardiac surgery, neuromuscular disorders, or presence of a cardiac pacemaker; Left ventricular ejection fraction <35%; mean pulmonary artery pressure >35 mmHg; Body mass index (BMI) <18 or >40; Emergency surgery; Hemodynamic instability (norepinephrine infusion >2 μg/kg/min) or intra-aortic balloon pump (IABP) support; Refractory hypotension or arrhythmias upon ICU admission; Postoperative bleeding >200 ml/h. Between March 2020 and March 2025, a total of 415 patients who underwent minimally invasive small-incision coronary artery bypass grafting were screened. and during this period, perioperative and ICU protocols remained consistent without any significant changes. Among them, 170 patients met the inclusion criteria and were ultimately included in the study. Patients were divided into the prone group and the supine group according to the recruitment strategy. 85 patients in the prone group and 95 in the supine group. To equalize the sample sizes, 85 patients were randomly selected from the supine group using simple random sampling. The detailed flow of patient selection is illustrated in Supplementary Figure S1.

This study was approved by the Ethics Committee of Beijing Chao-Yang Hospital, Capital Medical University (Approval No: 2023-Ke-673, Date: October 16, 2023).

### 2.2 Recruitment maneuver procedure

After enrollment, all patients were sedated with a continuous infusion of propofol to suppress spontaneous breathing. The period from enrollment to time zero was 30 min, during which randomization and baseline assessments were completed. At time zero, patients in the prone position group underwent both the prone positioning and the recruitment maneuver (15 min), followed by repositioning to supine (5 min), totaling 20 min. In the supine group, the recruitment maneuver was performed at the same time point, followed by a 20-min stabilization period. The procedure for turning the patient to prone and returning to supine was as follows: prior to prone positioning, patients were assessed to ensure no contraindications and hemodynamic stability was confirmed. Deep sedation was maintained (RASS ≤ −4). All vascular access lines, gastric tubes, and chest drains were checked for patency and secured. Pressure-prone areas, including the face, were protected using foam pads or skin protectors. Staff positioning was as follows: one at the head, one at the shoulders, and two at the hips. The mobility of all catheters was ensured, and the turning direction confirmed. For patients with ECMO or central venous catheters, the direction of turning followed the catheter pathway. The patient was turned to a 90° lateral position first. The head-end operator supported the patient's head and ensured catheter safety, then the team turned the patient fully into the prone position. The patient's face was gently positioned to the left or right on a sterile gauze or soft pad. The position of the endotracheal or tracheostomy tube was checked for dislodgement or compression. When repositioning to supine, the procedure was similar except no foam turning pad was needed. If cardiac arrest, severe hemodynamic instability, malignant arrhythmias, or airway device displacement occurred, the prone session was immediately terminated. The recruitment maneuver was performed as follows: before the maneuver, clinicians ensured the patient maintained hemodynamic stability (mean arterial pressure >70 mmHg) for at least 5 min with less than 10% fluctuation. The ventilator was set to pressure control mode, with a peak inspiratory pressure of 40 cmH_2_O, respiratory rate of 15 breaths/min, I:E ratio of 1:1, FiO_2_ of 0.8, and initial PEEP of 5 cmH_2_O. During the recruitment phase, PEEP was increased by 5 cmH_2_O every 20 s until a maximum of 20 cmH_2_O was reached and maintained for 40 s, totaling 100 s for the maneuver. If hemodynamic instability occurred (defined as a >40% drop in MAP), the maneuver was interrupted, and norepinephrine infusion was adjusted. Once hemodynamic stability was reestablished, the recruitment maneuver was resumed. A subsequent decremental PEEP trial was conducted to determine the optimal PEEP level. At the end of the final recruitment maneuver, PEEP was set to 20 cmH_2_O, and ventilation mode was switched to volume control: tidal volume 5–6 ml/kg, respiratory rate 15 breaths/min, I:E ratio 1:2, FiO_2_ of 0.8. PEEP was then decreased by 2 cmH_2_O every 15 s until the highest dynamic lung compliance was observed (i.e., before compliance began to decline). All procedures were performed by clinically trained physicians following the same protocol, ensuring consistency of recruitment maneuvers between the two groups. No patients in this study received extracorporeal membrane oxygenation (ECMO), and during the recruitment maneuvers, none of the patients experienced cardiac arrest, severe hemodynamic instability, malignant arrhythmias, or endotracheal tube dislodgement.

### 2.3 Data collection

Baseline patient information was collected, including sex, age, body mass index (BMI), and history of smoking, diabetes, hypertension, chronic renal failure, peripheral vascular disease, cerebrovascular accident, New York Heart Association (NYHA) functional classification, prior myocardial infarction, and prior percutaneous coronary intervention (PCI). Surgical type was also recorded, distinguishing between isolated MIDCABG and hybrid coronary revascularization (HCR, i.e., MIDCABG combined with PCI).

At the following time points—baseline (T0), immediately after completion of the recruitment maneuver (T1), 4 h after the maneuver (T2), and immediately following tracheal extubation after a spontaneous breathing trial (T3)—the following parameters were collected: PaO_2_/FiO_2_ ratio, peripheral capillary oxygen saturation (SpO_2_), arterial partial pressure of carbon dioxide (PaCO_2_), and heart rate (HR). During PaCO_2_ measurements, the minute ventilation was kept consistent within the two groups.

Left ventricular ejection fraction (LVEF), C-reactive protein (CRP), procalcitonin (PCT), and peripheral white blood cell (WBC) count were measured at baseline (T0) and at discharge (T5).

Personnel collecting physiological data were blinded to the intervention in order to minimize bias during data collection.

On the second day following the recruitment maneuver, bedside chest radiography was performed to assess pulmonary atelectasis (17, 18). Atelectasis was scored using a lobar scoring system, with a total score of 15 points; higher scores indicated more severe atelectasis. Each lobe (five lobes in total: right upper, right middle, right lower, left upper, and left lower) was scored individually as follows: 0 = no atelectasis, 1 = mild collapse (<50%), 2 = moderate collapse (50%–75%), and 3 = severe collapse (>75%). The total score was the sum of the five lobes, ranging from 0 to 15 (19, 20). Two experienced radiologists independently scored the images while blinded to the intervention group. In cases of disagreement, a third expert participated in discussion to reach consensus. Inter-rater agreement was assessed using Cohen's *κ*, and the Cohen's *κ* in this study was 0.77, indicating good consistency between the raters.

Patients with missing key variables (e.g., recruitment method) or with a high proportion of missing data (>30%) were excluded. For variables with a low proportion of missing data (<5%), median imputation was applied; for variables with a higher but still acceptable proportion of missing data (5%–30%), multiple imputation was performed.

### 2.4 Statistical analysis

All analyses in this study were performed using R software version 4.4.1. Continuous variables were expressed as medians (minimum–maximum) and compared using the Mann–Whitney *U* test or independent samples *t*-test. Categorical variables were presented as counts (percentages) and analyzed using Fisher's exact test or the chi-square test. A *post hoc* power analysis was conducted to assess the reliability of the results. For continuous variables, assuming a medium standardized effect size (Cohen's *d* = 0.5), with a sample size of *n* = 85 per group and a significance level of *α* = 0.05, the *post hoc* analysis indicated a statistical power of approximately 90%. For categorical variables, assuming a medium effect size (Cohen's *w* = 0.3) and a total sample size of *N* = 170, with a significance level of *α* = 0.05, the *post hoc* analysis indicated a statistical power of approximately 97%, suggesting that the observed results in this study are highly reliable. The improvement in oxygenation index was defined as the difference between the PaO_2_/FiO_2_ ratio at time point T3 and that at baseline (T0). Three multivariate linear regression models were constructed: Model 1 included only the recruitment maneuver strategy; Model 2 included age, sex, BMI, and recruitment maneuver strategy; Model 3 included all baseline characteristics along with the recruitment maneuver strategy. The dependent variable in all models was the degree of oxygenation index improvement. Variables other than the recruitment maneuver strategy that showed a significant effect on oxygenation improvement in the multivariate analysis were selected for subgroup stratification analysis, aiming to further explore the differences and robustness of the recruitment maneuver's effect on oxygenation improvement across different patient subgroups. The primary outcome of this study was the improvement in oxygenation index. The secondary outcomes included postoperative clinical recovery indicators (total length of hospital stay, ICU stay, time to extubation, time to first ambulation, time to first flatus, duration of invasive mechanical ventilation, and duration of noninvasive oxygen therapy), physiological parameters (peripheral capillary oxygen saturation, arterial partial pressure of carbon dioxide, heart rate, and left ventricular ejection fraction), inflammatory and laboratory markers (C-reactive protein, procalcitonin, and peripheral white blood cell count), and imaging findings (Lobar Atelectasis score).

## 3 Results

### 3.1 Baseline characteristics of the prone and supine groups

Overall, these characteristics showed no significant differences between the prone and supine groups, indicating good baseline balance and comparability (Table 1).

**Table 1:** Baseline differences between the prone position group and the supine position group.

Variables	All patients (*n* = 170)	Prone Group (*n* = 85)	Supine Group (*n* = 85)	*P*-value
Age	64 (52–78)	65 (52–78)	63 (52–77)	0.490
Gender				0.480
Male	127 (74.71%)	61 (71.76%)	66 (77.65%)	
Female	43 (25.29%)	24 (28.24%)	19 (22.35%)	
BMI	26.1 (19.7–32.1)	26.4 (19.8–31.9)	25.9 (19.7–32.1)	0.484
Smoking				0.107
Yes	59 (34.71%)	35 (41.18%)	24 (28.24%)	
No	111 (65.29%)	50 (58.82%)	61 (71.76%)	
Diabetes Mellitus				0.164
Yes	45 (26.47%)	18 (21.18%)	27 (31.76%)	
No	125 (73.53%)	67 (78.82%)	58 (68.24%)	
Hypertension				0.724
Yes	127 (74.71%)	62 (72.94%)	65 (76.47%)	
No	43 (25.29%)	23 (27.06%)	20 (23.53%)	
Chronic Renal Failure				0.129
Yes	4 (2.35%)	4 (4.71%)	0 (0%)	
No	166 (97.65%)	81 (95.29%)	85 (100%)	
Peripheral Vascular Disease				0.564
Yes	13 (7.65%)	8 (9.41%)	5 (5.88%)	
No	157 (92.35%)	77 (90.59%)	80 (94.12%)	
History of Cerebrovascular Accident				0.589
Yes	15 (8.82%)	9 (10.59%)	6 (7.06%)	
No	155 (91.18%)	76 (89.41%)	79 (92.94%)	
NYHA stage				0.617
NYHA I	64 (37.65%)	35 (41.18%)	29 (34.12%)	
NYHA II	88 (51.76%)	42 (49.41%)	46 (54.12%)	
NYHA III and IV	18 (10.59%)	8 (9.41%)	10 (11.76%)	
History of Myocardial Infarction				0.083
Yes	66 (38.82%)	27 (31.76%)	39 (45.88%)	
No	104 (61.18%)	58 (68.24%)	46 (54.12%)	
History of Percutaneous Coronary Intervention (PCI)				0.876
Yes	68 (40%)	33 (38.82%)	35 (41.18%)	
No	102 (60%)	52 (61.18%)	50 (58.82%)	
Whether PCI was performed concurrently during surgery				0.410
Yes	54 (31.76%)	24 (28.24%)	30 (35.29%)	
No	116 (68.24%)	61 (71.76%)	55 (64.71%)	

Continuous variables are presented as medians (range) and compared using the Mann–Whitney *U* or *t*-test. Categorical variables are expressed as counts (%) and analyzed with the chi-square or Fisher's exact test. A *P*-value <0.05 was considered statistically significant.

### 3.2 Postoperative clinical recovery differences between prone and supine groups

The median total hospital stay was 9 days (range: 7–12), with 9 days in the prone group and 10 days in the supine group, showing a statistically significant difference (*P* = 0.013). The median ICU stay was 27 h (range: 18–38 h), with no significant difference between the groups (*P* = 0.172). The median extubation time was 11 h overall, 11 h in the prone group, and 13 h in the supine group, with a significant difference (*P* = 0.034). The time to first ambulation was significantly earlier in the prone group (median 43 h vs. 51 h, *P* = 0.004). Time to first flatus was also earlier in the prone group (72 h vs. 78 h, *P* = 0.047). The duration of invasive mechanical ventilation was shorter in the prone group (21 h vs. 26 h, *P* = 0.004). The duration of non-invasive oxygen therapy was significantly shorter in the prone group compared to the supine group (63 h vs. 78 h, *P* < 0.001) (Table 2).

**Table 2:** Differences in postoperative clinical recovery indicators between the prone and supine groups.

Variables	All patients (*n* = 170)	Prone Group (*n* = 85)	Supine group (*n* = 85)	*P*-value
Length of hospital stay (days)	9 (7–12)	9 (7–12)	10 (7–12)	0.013
Length of ICU stay (hours)	27 (18–38)	27 (18–38)	29 (18–38)	0.172
Time to extubation (hours)	11 (6–16)	11 (6–16)	13 (6–16)	0.034
Time to first ambulation (hours)	49 (32–65)	43 (32–65)	51 (32–65)	0.004
Time to first flatus (hours)	77 (55–98)	72 (55–98)	78 (55–97)	0.047
Duration of invasive mechanical ventilation (hours)	24 (14–33)	21 (14–33)	26 (14–33)	0.004
Duration of noninvasive oxygen therapy	71 (47–93)	63 (47–93)	78 (47–93)	<0.001

Data are presented as median (range). Comparisons between prone and supine groups were performed using the Mann–Whitney *U* test. ICU, intensive care unit.

### 3.3 Differences in physiological and laboratory parameters between the prone and supine groups at different time points

#### 3.3.1 Primary outcome

**Figure 1:**
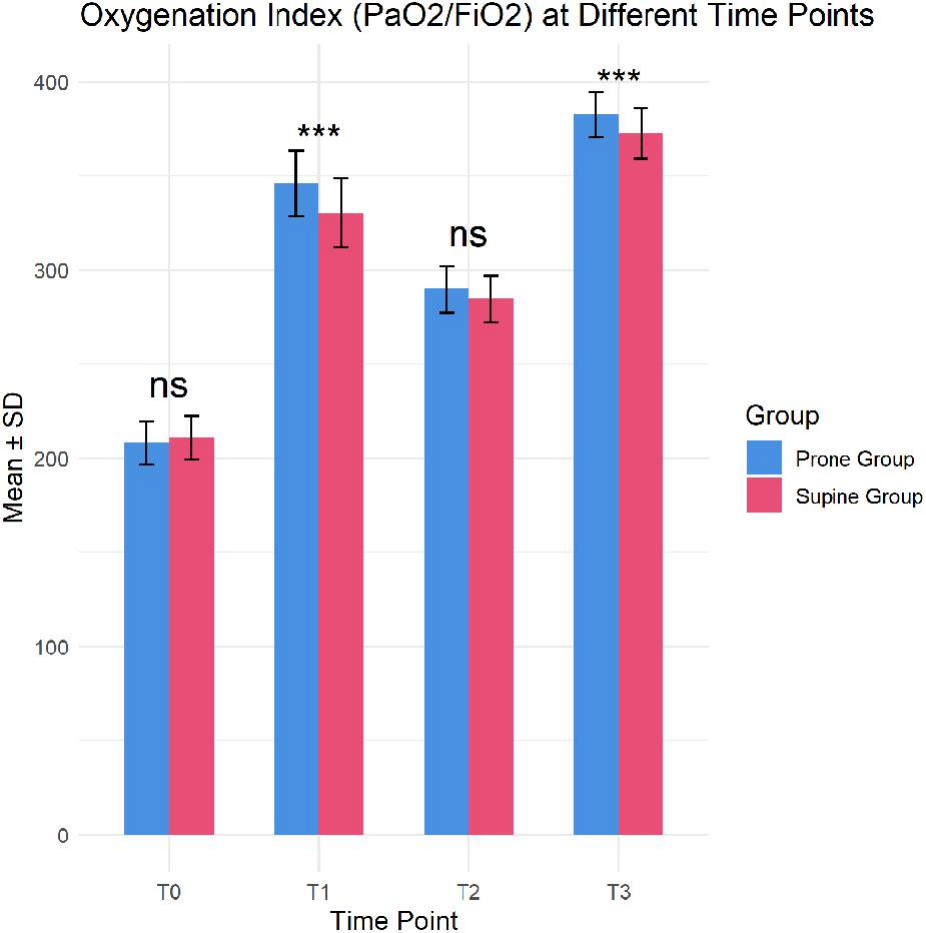
Differences in oxygenation Index between prone and supine groups at different time points (*p < 0.05; **p < 0.01; ***p < 0.001; ns, not significant).

There was no significant difference in the oxygenation index (PaO_2_/FiO_2_) between the two groups before the recruitment maneuver (T0) (206.3 vs. 211.0, *P* = 0.073). However, immediately after the maneuver (T1) and immediately after extubation (T3), the prone group showed significantly higher PaO_2_/FiO_2_ ratios than the supine group (T1: 354.5 vs. 322.5, *P* < 0.001; T3: 386.7 vs. 366.3, *P* < 0.001), suggesting that the prone recruitment strategy is more effective at improving oxygenation. At 4 h post-maneuver (T2), the difference was not statistically significant (*P* = 0.069) (Figure 1).

#### 3.3.2 Secondary outcome

The median peripheral capillary oxygen saturation (SpO_2_) was above 90% at all time points in both groups, with the prone group showing significantly higher SpO_2_ at T1, T2, and T3 (*P* = 0.033, 0.017, and 0.013, respectively). Arterial carbon dioxide partial pressure (PaCO_2_) at T1, T2, and T3 was significantly lower in the prone group compared to the supine group (all *P* ≈ 0.023), indicating better CO_2_ elimination in the prone position. Heart rate (HR) was significantly lower in the prone group than in the supine group at T1, T2, and T3 (all *P* < 0.05), with no difference before the maneuver (T0). Left ventricular ejection fraction (LVEF) was comparable between groups before the maneuver (T0) (51.8% vs. 52.7%, *P* = 0.269), but slightly higher in the prone group at discharge (T5) (55.3% vs. 54.2%, *P* = 0.029). C-reactive protein (CRP) levels showed no difference preoperatively (T0) but were significantly lower in the prone group at discharge (T5) (11.4 mg/L vs. 12.6 mg/L, *P* = 0.041), indicating reduced inflammation. Procalcitonin (PCT) levels were similar at baseline, with the prone group slightly lower at discharge (0.19 vs. 0.23 ng/ml, *P* = 0.034). Peripheral blood white blood cell count (WBC) showed no difference before the maneuver but was significantly lower in the prone group at discharge (6.0 vs. 7.2 × 10^9^/L, *P* = 0.021). Lobar Atelectasis scores showed no significant differences before the maneuver and on the second postoperative day, although overall atelectasis scores decreased (Table 3). These findings suggest that recruitment maneuvers significantly improve oxygenation, CO_2_ elimination, heart rate control, and inflammatory markers, with the prone position showing superior effects compared to the supine position.

**Table 3:** Differences in physiological and laboratory parameters between the prone and supine groups at different time points.

Parameters	All patients (*n* = 170)	Prone Group (*n* = 85)	Supine Group (*n* = 85)	*P*-value
PaO_2_/FiO_2_				
T0	208.1 (187.5–234.0)	206.3 (187.5–233.1)	211.0 (188.0–234.0)	0.073
T1	339.9 (290.4–383.8)	354.5 (290.4–383.4)	322.5 (291.5–383.8)	0.001
T2^a^	286.6 (270.2, 303.0)	292.0 (275.6, 308.4)	282.0 (265.7, 298.3)	0.069
T3	378.1 (345.1–410.3)	386.7 (346.1–410.3)	366.3 (345.1–410.0)	<0.001
Peripheral capillary oxygen saturation, SpO_2_ (%)				
T0	91.2 (88.3–94.0)	91.3 (88.4–94.0)	91.1 (88.3–94.0)	0.693
T1^a^	97.0 (95.7, 98.3)	97.2 (95.9, 98.5)	96.5 (95.2, 97.8)	0.033
T2^a^	95.8 (94.2, 97.4)	96.3 (94.8, 97.8)	95.5 (93.9, 97.1)	0.017
T3	97.6 (95.8–99.8)	98.0 (95.8–99.8)	97.3 (95.8–99.8)	0.013
Arterial partial pressure of carbon dioxide, PaCO_2_ (mmHg)				
T0	50.5 (47.5–54.1)	50.8 (47.5–54.0)	50.3 (47.6–54.1)	0.321
T1	44.4 (41.4–48.5)	44.0 (41.4–48.5)	44.9 (41.5–48.4)	0.023
T2	47.4 (44.6–50.1)	47.0 (44.6–50.0)	47.8 (44.7–50.1)	0.023
T3^a^	42.0 (39.4, 44.6)	41.2 (38.7, 43.7)	42.8 (40.2, 45.4)	0.027
Heart Rate, HR (bpm)				
T0	98 (82–113)	96 (82–113)	100 (84–113)	0.130
T1	89 (77–102)	85 (77–102)	94 (77–102)	0.001
T2^a^	93 (84, 102)	88 (79, 97)	97 (88, 106)	0.001
T3	89 (75–99)	87 (75–99)	90 (76–99)	0.032
Left Ventricular Ejection Fraction, LVEF (%)				
T0	52.6 (46.5–58.1)	51.8 (46.6–58.1)	52.7 (46.5–58.1)	0.269
T5	54.9 (49.6–61.6)	55.3 (49.9–61.2)	54.2 (49.6–61.6)	0.029
C-Reactive Protein, CRP (mg/L)				
T0	62.6 (35.6–89.5)	61.9 (36.6–89.5)	62.7 (35.6–88.2)	0.843
T5	12.0 (8.7–15.8)	11.4 (8.9–15.8)	12.6 (8.7–15.8)	0.041
Procalcitonin, PCT (ng/ml)				
T0	0.60 (0.09–1.13)	0.58 (0.11–1.13)	0.62 (0.09–1.13)	0.648
T5	0.21 (0.03–0.38)	0.19 (0.03–0.38)	0.23 (0.04–0.37)	0.034
Peripheral White Blood Cell count, WBC (×10^9^/L)				
T0	12.9 (10.2–15.9)	12.9 (10.3–15.8)	13.5 (10.2–15.9)	0.265
T5	6.5 (4.5–8.9)	6.0 (4.6–8.8)	7.2 (4.5–8.9)	0.021
Lobar Atelectasis Score				
T0	5 (3–8)	5 (3–8)	5 (3–8)	0.904
The second day after lung recruitment maneuver	3 (1–4)	2 (1–4)	3 (1–4)	0.300

a Indicates that the data were analyzed using independent samples *t*-test and presented as mean with 95% confidence interval (CI).

The remaining unmarked data were analyzed using Mann–Whitney *U* test and presented as median (range). With *P* < 0.05 considered statistically significant. T0, baseline; T1, immediately after the recruitment maneuver post-lung recruitment; T2, 4 h post-recruitment maneuver; T3, immediately after extubation following the spontaneous breathing trial; and T5, at discharge.

### 3.4 Multivariate linear regression analysis of the effect of recruitment maneuver strategy on oxygenation improvement

Model 1, including only the recruitment maneuver strategy, showed a significant effect on oxygenation improvement, with a regression coefficient of 15.275 (standard error 3.997), *t*-value 3.821, and *P* < 0.001, indicating a significant positive effect of the prone recruitment strategy on oxygenation improvement. In Model 2, after adjusting for age, sex, and BMI, the recruitment maneuver strategy remained significant (estimate: 15.246, *P* < 0.001), while age, sex, and BMI had no significant effects (all *P* > 0.1). In Model 3, which included all baseline characteristics such as smoking history, diabetes mellitus (DM), and hypertension, the recruitment maneuver strategy continued to significantly affect oxygenation improvement (estimate: 15.282, *P* < 0.001). Non-diabetic patients showed significantly greater oxygenation improvement than diabetic patients (estimate: −8.429, *P* = 0.043), and a history of PCI was also associated with a significantly negative effect on oxygenation improvement (estimate: −7.183, *P* = 0.049) (Table 4).

**Table 4:** Multivariate linear regression analysis of the effect of lung recruitment strategies on the oxygenation index.

Model	Term	Estimate	Std. error	Statistic	*p* value
Model 1	Method	15.275	3.997	3.821	< 0.001
Model 2	Method	15.246	4.009	3.803	<0.001
	Age	−0.212	0.261	−0.810	0.419
	Gender	−6.912	4.611	−1.499	0.136
	BMI	−0.547	0.571	−0.957	0.340
Model 3	Method	10.745	4.322	2.486	0.014
	Age	0.062	0.275	0.225	0.822
	Gender	−4.266	4.990	−0.855	0.394
	BMI	−0.390	0.614	−0.636	0.526
	Smoking	−5.556	4.608	−1.206	0.230
	Diabetes Mellitus	5.103	4.906	1.040	0.300
	Hypertension	−4.297	5.082	−0.846	0.399
	Chronic Renal Failure	−10.785	13.967	−0.772	0.441
	Peripheral Vascular Disease	7.358	7.850	0.937	0.350
	History of Cerebrovascular Accident	6.280	7.661	0.820	0.414
	NYHA stage	4.387	3.366	1.304	0.194
	History of Myocardial Infarction	−5.974	4.398	−1.358	0.176
	History of Percutaneous Coronary Intervention (PCI)	−3.788	4.286	−0.884	0.378
	Concomitant PCI	11.366	4.622	2.459	0.015

### 3.5 Stratified analysis

**Figure 2:**
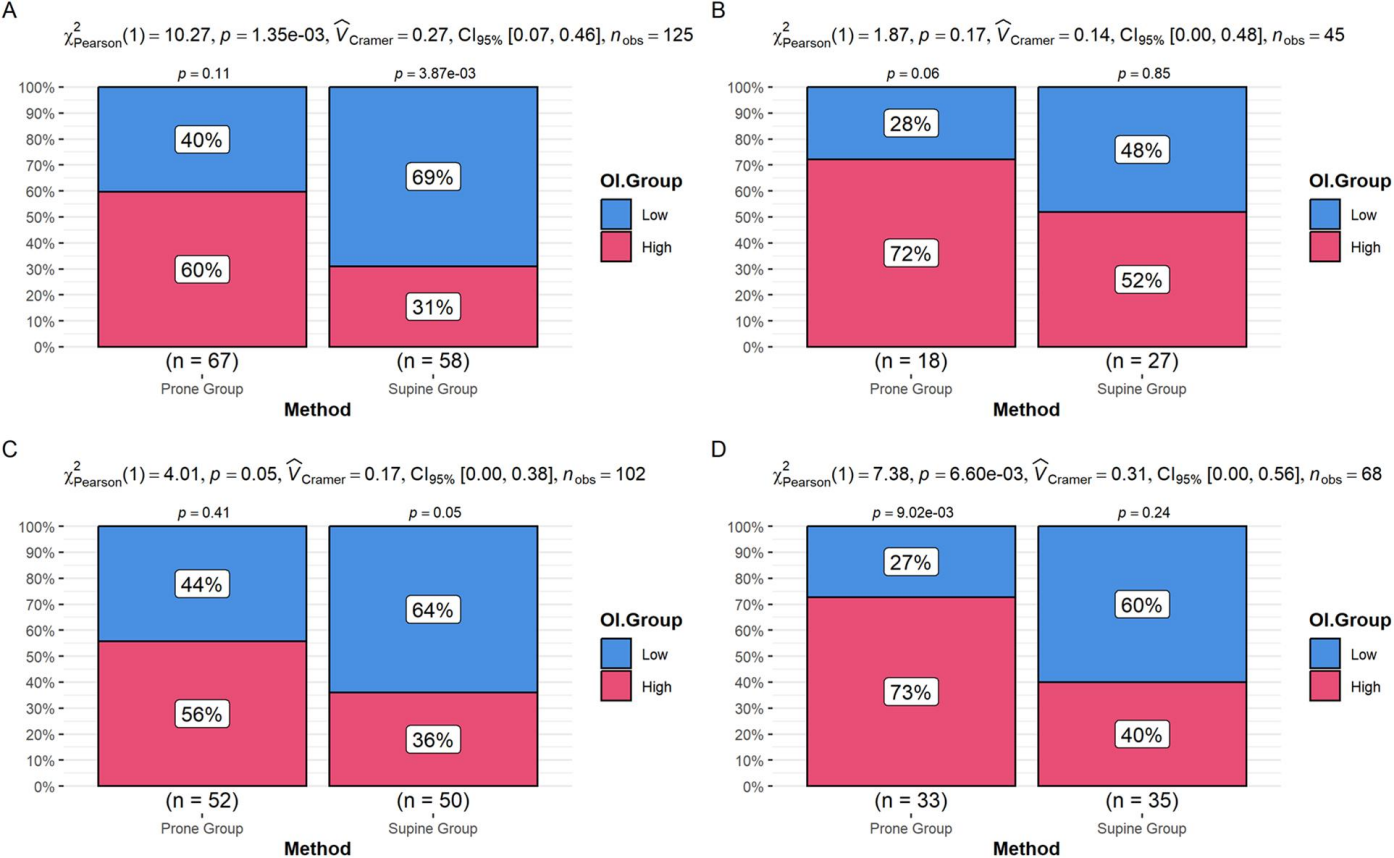
**(A)** Difference in the proportion of oxygenation index improvement between the prone and supine groups in non-diabetic patients. **(B)** Difference in the proportion of oxygenation index improvement between the prone and supine groups in diabetic patients. **(C)** Difference in the proportion of oxygenation index improvement between the prone and supine groups in patients without a history of PCI. **(D)** Difference in the proportion of oxygenation index improvement between the prone and supine groups in patients with a history of PCI.

The above multivariate linear regression analysis indicated that diabetes mellitus and a history of PCI also had significant impacts on the degree of improvement in the oxygenation index; they may act as effect modifiers or important confounders. Therefore, we further performed stratified analyses by diabetes status and PCI history to explore the effects of different recruitment maneuvers on oxygenation improvement within these subgroups. The results showed that in the non-diabetic group, the proportion of patients with significant oxygenation improvement was markedly higher in the prone group compared to the supine group, while no significant difference was observed in the diabetic group (Figures 2A,B). In both the non-PCI and PCI history groups, the prone group exhibited a significantly higher proportion of oxygenation improvement than the supine group, with a stronger significance observed in the PCI history group (Figures 2C,D). The interaction analysis in the multivariate regression model also shows that the interaction term between the recruitment method and diabetes has an OR of 0.383 (95% CI: 0.167–0.811). The interaction term between the recruitment method and prior PCI has an OR of 3.115 (95% CI: 1.489–6.516) (Table 5). This suggests that in non-diabetic patients and those with prior PCI, the prone recruitment strategy is more strongly associated with improvement in the oxygenation index. However, these analyses are preliminary and exploratory, and further validation and confirmation are needed.

**Table 5:** Interaction analysis.

Term	Estimate	Std. error	Statistic	*p* value	OR	CI_lower	CI_upper
(Intercept)	−0.799	0.284	−2.813	0.005	0.450	0.258	0.785
Method	1.192	0.378	3.155	0.002	3.292	1.571	6.901
DM	0.873	0.478	1.824	0.068	2.393	0.937	6.113
Method*DM	−0.961	0.423	−2.270	0.023	0.383	0.167	0.811
(Intercept)	−0.575	0.295	−1.953	0.051	0.563	0.316	1.002
Method	0.807	0.406	1.989	0.047	2.242	1.012	4.967
PCI	0.170	0.454	0.374	0.708	1.185	0.487	2.884
Method*PCI	1.136	0.377	3.017	0.003	3.115	1.489	6.516

Diabetes is coded as 1 for diabetic patients and 0 for non-diabetic patients. A history of PCI treatment is coded as 1 for patients with PCI history and 0 for those without. High improvement in oxygenation index is coded as 1, and low improvement in oxygenation index is coded as 0.

## 4 Discussion

Our results indicate that recruitment maneuvers were associated with improvements in patients' oxygenation, carbon dioxide elimination, heart rate control, and inflammatory markers, with prone positioning showing more favorable effects compared to supine. This may be attributed to recruitment maneuvers reopening partly dorsally collapsed alveoli by increasing inspiratory airway positive pressure and PEEP, effectively expanding ventilated lung areas and improving ventilation/perfusion (V/Q) matching (21, 22).

### 4.1 Prone position and recruitment vs. supine

Prone recruitment is associated with enhanced ventilation of the dorsal lung regions and with reductions in atelectasis and hypoventilated areas, which may contribute to more optimal alveolar ventilation–perfusion matching and improved oxygenation (16). The dorsal lung is more prone to collapse and perfusion excess in the supine position. Additionally, in the supine position, the lung bases bear greater pressure, whereas prone positioning, with a more homogenous ventilation, resulting in more uniform stress and strain, reduced localized alveolar stress, decreased risk of lung injury, and improved lung compliance (23). Atelectasis leads to regions with normal perfusion but absent ventilation, causing shunt and V/Q mismatch (24); Recruitment reopens these areas, and prone recruitment further reduces overventilation of anterior alveoli while maintaining perfusion of the dorsal regions, promoting more efficient gas exchange and CO_2_ elimination (25). Recruitment maneuvers are associated with improved lung compliance and reduced work of breathing; in the prone position, the chest and abdominal structures exert less diaphragmatic pressure, allowing more natural lung expansion, increased tidal volume, decreased respiratory rate, and improved CO_2_ clearance per breath (26).

In addition, during prone lung recruitment, the lungs operate between “two rigid bars.” This arrangement allows stress on the alveoli at end-inspiration and end-expiration to be more evenly distributed, reducing localized alveolar overdistension and collapse, thereby improving lung compliance and gas exchange (27). Improvement in oxygenation and ventilation efficiency was still observed at the subsequent time point (T3), indicating that the beneficial effects of lung recruitment persist even after turning the patient supine.

### 4.2 Oxygenation and sympathetic activity

Patients with atelectasis often experience hypoxemia, which strongly stimulates sympathetic nervous activity and increases heart rate (28). By improving oxygenation, recruitment maneuvers alleviate hypoxemia and suppress sympathetic overactivity, naturally reducing heart rate. Since prone recruitment is generally linked with greater improvements in oxygenation, it also shows superior heart rate control. Elevated PaCO_2_ stimulates brainstem chemoreceptors (29), increasing sympathetic tone and heart rate; recruitment promotes CO_2_ elimination, alleviating hypercapnia-induced tachycardia. With better lung compliance, reduced dead space, and higher ventilation efficiency in the prone position, heart rate regulation is more pronounced. Lower heart rate suggests alleviated postoperative sympathetic overactivation, stabilizing circulation, essential for reducing ICU stay and promoting early rehabilitation.

### 4.3 Inflammation

Recruitment maneuvers, especially in the prone position, were associated with reductions in postoperative inflammatory markers. Atelectasis-induced hypoxia activates inflammatory pathways via hypoxia-inducible factor-1α (HIF-1α) (30), increasing pro-inflammatory cytokines such as IL-6 and TNF-α. By improving oxygenation, recruitment reduces hypoxia-related inflammation, lowering CRP and PCT levels. prone recruitment improves ventilation of deeper lung regions, leading to greater oxygenation and stronger anti-inflammatory effects. Repeated collapse and reopening of atelectatic areas cause shear stress injury to alveoli, releasing inflammatory mediators; recruitment stabilizes alveoli, reducing injury, and prone recruitment distributes airway pressures more evenly, further mitigating localized stress and inflammation. Decreased inflammatory markers imply reduced pulmonary and systemic inflammation, aiding gastrointestinal recovery and shortening time to first flatus.

### 4.4 Impact of respiratory variables

Our study found that prone recruitment was associated with shorter extubation time, reduced duration of mechanical ventilation, decreased length of hospital stay, and earlier mobilization and gastrointestinal function recovery. These findings may be related to improvements in oxygenation, CO_2_ elimination, heart rate control, and inflammation reduction. Higher oxygenation index (PaO_2_/FiO_2_) was associated with earlier weaning and extubation, while decreased PaCO_2_ reflected better lung ventilation efficiency and reduced respiratory workload, corresponding to shorter invasive and non-invasive ventilation durations. After adjusting for comprehensive baseline covariates, prone recruitment remained the only stable and significant factor associated with oxygenation improvement, suggesting its potential independent and consistent therapeutic benefit across patients with different comorbidities, body habitus, and age. These results indicate clinical reproducibility and stability, supporting its potential value for broader application. Since the application of this method in cardiac surgery is relatively novel, future prospective studies and randomized controlled trials are needed to confirm these findings and explore the long-term benefits of prone recruitment for a broader patient population in cardiac surgery. Ultimately, incorporating the prone recruitment strategy into routine postoperative care may become a promising alternative or adjunct to standard respiratory support.

### 4.5 Stratified analysis

Stratified analysis suggested diabetes might attenuate the effect of prone recruitment, possibly due to diabetes-induced changes in capillary permeability and alveolar-capillary barrier damage, limiting oxygenation improvement. Elevated inflammatory cytokines in diabetes may impair V/Q matching restoration after recruitment, and reduced lung compliance may hinder recruitment efficacy. This highlights the need for more potent or individualized recruitment strategies in diabetic patients. Conversely, prior PCI history enhanced the effect of prone recruitment, potentially because these patients often have left ventricular dysfunction or elevated pulmonary pressures causing uneven lung perfusion and V/Q mismatch. Prone recruitment improves ventilation and perfusion distribution in dorsal lung regions, significantly enhancing V/Q matching and oxygenation, with greater benefit compared to patients without prior PCI who have relatively normal pulmonary blood flow.

### 4.6 Limitations

This study has several limitations. First, it is a retrospective cohort study, which may be subject to selection bias and unmeasured confounding factors. Second, it is a single-center study with a relatively small sample size, which may limit the generalizability of the findings. Third, although simple random sampling was applied to the supine group to balance the sample size, complete randomization was not achieved, and systematic differences may still exist. Fourth, prone and supine recruitment maneuvers may have been performed at different time points; despite the use of a standardized protocol, variations in the operating environment or other factors could still affect the outcomes. Fifth, this study relied on clinical observational data and did not investigate the detailed physiological mechanisms underlying improvements in oxygenation, CO_2_ elimination, heart rate control, and inflammatory response. Finally, long-term outcomes, such as pulmonary function or cardiovascular events, were not assessed. Future large-scale, multicenter, prospective randomized controlled trials are needed to validate the efficacy of prone recruitment, explore its mechanisms, and evaluate long-term effects.

## 5 Conclusion

In patients with atelectasis following MIDCABG, lung recruitment maneuvers were observed to be associated with improvements in oxygenation, carbon dioxide elimination, heart rate control, and inflammatory markers, with prone-position recruitment showing relatively more pronounced effects. These improvements may contribute to clinical recovery. After adjusting for multiple baseline variables, prone recruitment remained consistently associated with the degree of oxygenation improvement, suggesting its potential applicability across different patient populations. The data also indicate that the magnitude of improvement may be lower in patients with diabetes, whereas it may be higher in those with a history of PCI. Future large-scale prospective studies are needed to validate these observations and further explore individualized recruitment strategies.

## Data Availability

The original contributions presented in the study are included in the article/Supplementary Material, further inquiries can be directed to the corresponding author.
