# Supplementary figures and images for: Study on the application of prone and supine lung recruitment maneuvers in the treatment of atelectasis after minimally invasive direct coronary artery bypass surgery
Source: Front Surg. 2025 Oct 2;12:1665139. doi: 10.3389/fsurg.2025.1665139 (PMC12528020; doi:10.3389/fsurg.2025.1665139)

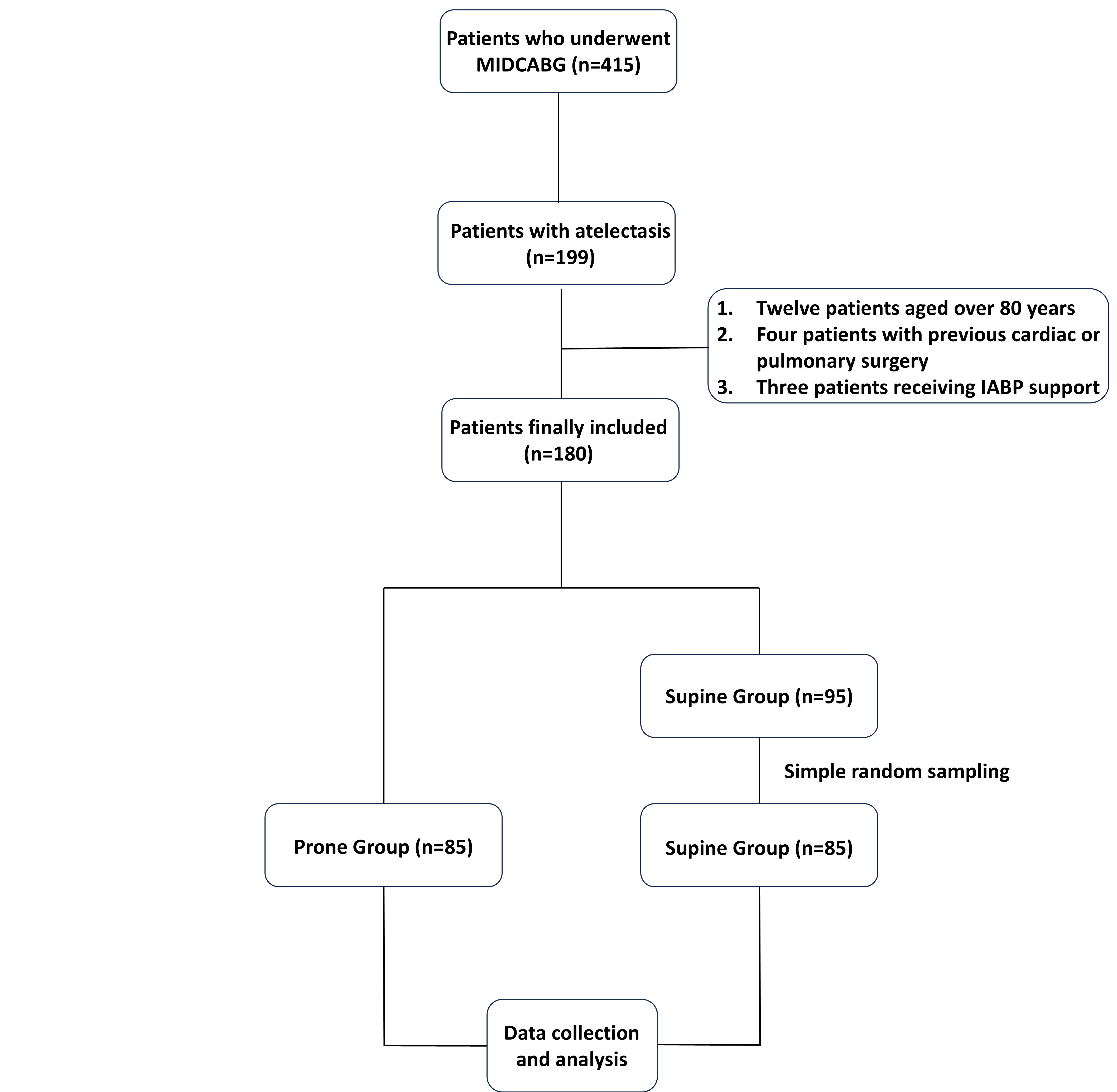

Supplement: Supplementary file 1 [file Image1.tif]
